# Characterization of Shiga Toxin-Producing *Escherichia coli* Isolated from Ground Beef Collected in Different Socioeconomic Strata Markets in Buenos Aires, Argentina

**DOI:** 10.1155/2014/795104

**Published:** 2014-06-09

**Authors:** Patricia Llorente, Laura Barnech, Kinue Irino, María Valeria Rumi, Adriana Bentancor

**Affiliations:** ^1^Microbiología, Facultad de Ciencias Veterinarias, Universidad de Buenos Aires, Chorroarín 280, CP1427CWO Buenos Aires, Argentina; ^2^Instituto Adolfo Lutz, 01246-000 Sao Paulo, SP, Brazil

## Abstract

Consumption of raw/undercooked ground beef is the most common route of transmission of Shiga toxin-producing *E. coli* (STEC). The aim of the study was to determine the STEC contamination level of the ground beef samples collected in 36 markets of different socioeconomic strata in Buenos Aires, Argentina, and the characterization of the isolated strains. Ninety-one out of 252 (36.1%) samples were *stx+*. Fifty-seven STEC strains were recovered. Eleven STEC strains belonged to O157 serogroup, and 46 to non-O157 serogroups. Virulence markers of the 57 STEC were *stx*1, 5.3% (3/57); *stx*2, 86.0% (49/57); *stx*1/*stx*2, 8.8% (5/57); *ehx*A, 61.4% (35/57); *eae*, 26.3% (15/57); *saa*, 24.6% (14/57). Shiga toxin subtypes were *stx*2, 31.5% (17/54); *stx*2c-vhb, 24.1% (13/54); *stx*2c-vha, 20.4% (11/54); *stx*2/*stx*2c-vha, 14.8% (8/54); *stx*2/*stx*2c-vhb, 5.6% (3/54); *stx*2c-vha/vhb, 3.7% (2/54). Serotypes O178:H19 and O157:H7 were prevalent. Contamination rate of STEC in all strata was high, and the highest O157 contamination was observed at low strata at several sampling rounds. Persistence of STEC was not detected. Sixteen strains (28.1%) were resistant to ampicillin, streptomycin, amikacin, or tetracycline. The STEC contamination level of ground beef could vary according to the sociocultural characteristics of the population.

## 1. Introduction

Shiga toxin, a potent cytotoxin, is mainly produced by* Escherichia coli* and* Shigella dysenteriae* type 1, and sporadically it can also be produced by* Citrobacter freundii*,* Enterobacter cloacae*,* Shigella flexneri*,* Shigella sonnei*,* Aeromonas hydrophila*, and* Aeromonas caviae* [1–3].

Shiga toxin-producing* E. coli* (STEC) strains can produce two types of toxins, Stx1 and Stx2, and are the main causes of hemorrhagic colitis (HC) and hemolytic uremic syndrome (HUS) [4, 5]. There is evidence that the association between Stx2 and the presence of intimin (*eae*), a specific adhesin, is a predictor of HUS [6].

Unlike the commensal* E. coli *strains, STEC strains have several virulence genes (*stx*1,* stx*2,* eae*,* ehx*A,* saa*) which permit the evaluation of their pathogenic nature in the laboratory [7, 8]. Ruminants, especially cattle, constitute a vast reservoir of STEC, and it is not surprising that human infection can frequently be traced to contamination of food or water with cattle feces [9].

More than 400 serotypes of STEC were identified. Although the prototype associated with the major proportion of HUS cases is STEC O157:H7, non-O157 group was identified to be at risk of HUS. The strains of non-O157 were classified in seropathotypes according to their relation with severity and their frequency as a cause of large outbreaks [11].

In Argentina many outbreaks of nonbloody diarrhea, bloody diarrhea, and HUS associated with O157 and non-O157 STEC infections have been identified through the national surveillance system. Consumption of raw/undercooked ground beef is the most common means of transmission of STEC. Several reports in Argentina showed different prevalence of STEC in meat products [12–15]. The implication of the isolation of STEC from food can be assessed by serotypes categorization, virulence profile, and antibacterial susceptibility [16, 17]. The STEC contamination level of ground beef could vary due to the sociocultural characteristics of the population [18].

The purpose of this study was to determine the STEC contamination level of the ground beef samples, obtained from markets with different socioeconomic profiles in Buenos Aires, Argentina, and to characterize the virulence profiles and the antimicrobial susceptibility patterns of the strains.

## 2. Materials and Methods

### 2.1. Sample Collection

Ground meat samples were bought from markets in San Martín district, an urban area of Buenos Aires, during Spring and Summer from 2006 to 2009. This district is located at the urban ring of Buenos Aires City having the five socioeconomic levels.

The study was stratified by socioeconomic level. Documentary information was obtained from the Instituto Nacional de Estadísticas y Censos (INDEC—National Institute of Statistics and Census), which defines the socioeconomic levels of the district of San Martín as high, medium-high, medium, medium-low, and low [19]. For this study, the five socioeconomic levels (INDEC) were partially combined to create three strata: high (high and medium-high), medium (medium), and low (medium-low and low) [20]. Markets representing 30% of each socioeconomic stratum were selected by simple random sampling. Thirty-six markets, 11 from high stratum (HS), 13 from media stratum (MS), and 12 from low stratum (LS), were sampled in the same epidemiological week by seven rounds.

A total of 252 samples were collected in sterile bags avoiding contamination among them and were immediately transported to the laboratory in cooled boxes.

### 2.2. Cultures

One portion of each sample (65 g) was stomached in 585 mL of Tryptic Soy Broth (TSB) for 2 minutes for screening for STEC non-O157 group and another portion of each sample (65 g) was stomached in 585 mL of TSB with novobiocin plus casaminoacids (mTSB+n) [21] for 2 minutes for screening for STEC O157 serogroup. The broths were incubated at 37°C or 42°C, respectively. Cultures from TSB were streaked onto MacConkey agar (MAC), and 1 mL from mTSB+n was tested for O157:H7 by immunomagnetic separation (IMS) (Neogen's immunobeads) and streaked onto sorbitol MacConkey-CT agar (SMAC-CT). Plates were incubated in aerobiosis at 37°C for 18 hours.

### 2.3. Screening for STEC Strains by PCR

*E. coli* strains ATCC 25922 (*stx*1-,* stx*2-,* eae*-,* saa*-,* ehx*A-, and* rfb*O157*-*), EDL 933 (O157:H7,* stx*1*+*,* stx*2*+*,* eae+*,* saa-*,* ehx*A*+* and* rfb*O157*+*), and UNCPBA O91:H21 (*stx*1*+, stx*2*+, eae-, saa+, ehx*A*+* and* rfb*O157-) were used as controls. Screening for* stx*1,* stx*2, and* rfb*O157 genes was performed by PCR as previously described from the confluence zone of MAC and SMAC-CT plates as template [22]. From each positive plate, isolation of a representative strain was investigated in up to 50 CFU through pools with up to ten colonies. Individual colonies of each positive pool were retested by PCR. The STEC isolated was kept in TSB + glycerol (20%) at −196°C. For further identification, all positive colonies to any of the genes under study were streaked on Tryptic Soy Agar plates.

### 2.4. Characterization of STEC Strains

All strains were confirmed as* E. coli* by biochemical tests as previously described [22] and the enterohemolytic activity was determined using washed sheep blood cells agar supplemented with calcium according to Beutin et al. [23]. Identification of somatic (O) and flagellar (H) antigens was performed following standard methods of tube agglutination test [24] and using currently available O (O1 to O181) and H (H1 to H56) antisera as described elsewhere [25]. All strains carrying the* stx* genes were tested by PCR for the presence of* eae*,* saa*, and* ehx*A virulence genes. Primers and PCR conditions were previously described [7, 8, 22]. Genotyping of* stx*2 variants was performed by RFLP-PCR according to previous reports using primers VT2-c/VT2-d, VT2v-1/VT2v-2 [26], VT2-e/VT2-f [27], and SLTv-IIvc/CKS-2 [28]. Strains identified as mucus-activatable* stx*2d variant at screening were then analyzed by the one-step PCR method described by Zheng et al. [10].

### 2.5. Antimicrobial Susceptibility

Antimicrobial susceptibility patterns were determined by disk diffusion susceptibility tests in Mueller Hinton Agar (Britania) according to the Performance Standards for Antimicrobial Susceptibility Testing [29]. Firstly, we used the following antimicrobials (BBL): penicillins: ampicillin (AMP); aminoglycosides: amikacin (AK), gentamicin (CN), and streptomycin (S); quinolones: nalidixic acid (NA) and ciprofloxacin (CIP); chloramphenicol (C), nitrofurantoin (F), tetracycline (TE), and trimethoprim-sulfamethoxazole (SXT) according to Argentinean consensus for* Enterobacteriaceae* [30].* E. coli* ATCC 25922 was used as the control strain. The strains were reported as susceptible (S), intermediate—having reduced susceptibility—(r), or resistant (R) [29, 30].

The characterization of multiresistant strains was done when coresistance to four or more unrelated families of antimicrobials was detected.

Secondly, all the strains R/r to AMP were evaluated for *β*-lactamases detection using the following antibacterial agents (BBL): amoxicillin clavulanic acid (AMC), amoxicillin sulbactam (SAM), aztreonam (ATM), cefepime (FEP), cefotaxime (CTX), cefotaxime/clavulanic acid (CTX/CLA), cefoxitin (FOX), cefpodoxime (CPD), ceftriaxone (CRO), cephalothin (KF), imipenem (IPM), meropenem (MEM), piperacillin (PRL), and piperacillin tazobactam (TZP).* E.coli* 35218 was used as the control strain [29].

The screening tests for extended spectrum *β*-lactamases (ESBL) were evaluated by ATM, CTX, CPD, and CRO diameters [29, 30] and confirmed by ≥5 mm diameters increase by CTX/CLA compared with CTX alone. Screening test for carbapenemases was evaluated with cephalosporin class III CTX and CRO [29].

### 2.6. Study of Strain Persistence at Outlets Over Time

Thirty-six outlets were evaluated for contamination persistence. Identity of STEC strains was determined through subtyping. The used algorithm included sequential steps of serotyping,* stx* subtypes, virulence factors characterization, biotype, and antibiotype through antimicrobial susceptibility pattern. In case of strains showing identity at phenotype and virulence profile evaluated, a high discriminatory power technique, PFGE, was proposed to confirm the relation between isolates.

### 2.7. Statistical Analysis

Statistical analysis data was processed by the program EpiInfo 2002 version 3.2 (CDC-WHO). The statistical analysis of the variables under study, as classified by socioeconomic strata, was performed using Yates corrected Chi-square independence test and the *Z* test to compare two proportions. Statistical significance was regarded at a *P* value <0.05 [31].

## 3. Results

A total of 91 out of 252 (36.1%) screened samples were positive for one of the* stx* genes. The distribution of positive samples according to their strata was as follows: 25/77 (32.5%, CI 95% 21.36–43.58) from HS, 35/91 (38.46%, CI 95% 27.92–49.01) from MS, and 31/84 (36.90%, CI 95% 25.99–47.82) from LS.

The distribution of markets, according to the socioeconomic strata in which at least one sample was positive for the* stx* genes was 8/11 (72.7%) from HS, 10/13 (76.9%) from MS, and 10/12 (83.3%) from LS.

Among the 91 positive samples by screening, 57 STEC strains were recovered: 11 STEC O157 and 46 STEC non-O157.

Serotyping of the STEC isolates showed that the 57 strains belonged to 14 different O-groups and 10 different H antigens were identified as well nontypeable (ONT) and nonmotile (NM) strains (Table 1).

Among the 21 non-O157 serotypes, O178:H19 (13) and O174:H21 (3) prevailed. Interestingly, from four samples two different serotypes were recovered, which were three from MS: (i) O178:H19 and O174:H21; (ii) O178:H19 and ONT:H8; (iii) O2:H25 and O26:H11 and one from LS: (iv) O157:H7 and ONT:H19 (Table 1).

Among eleven O157 strains, 10 were O157:H7, one was O157:NM, and all of them were sorbitol nonfermenting and *β*-glucuronidase-negative.

Both prevalent serogroups, O157 and O178, were isolated from the three strata (Table 1).

In total STEC O157 was recovered in 6/7 rounds of sampling. Interestingly, their distribution was only 1 strain from HS, 5 strains from MS in 3 (42.8%) of the rounds in 4 different markets, and 5 strains from LS in 5 (71.4%) of the rounds in 3 different markets (Tables 1 and 2).

On the other hand, the prevalent O178:H19 serotype was recovered in 5 sampling rounds, and surprisingly, 6 strains were from the first round of HS and MS with 3 strains each. In total 5 strains were from HS, 6 from MS, and 2 from LS.

Despite the results that showed that contamination by STEC in some markets was recurrent up to 4 rounds of sampling (Table 1), identity among isolations was not maintained. Even though STEC O157 with similar virulence profiles was isolated more than once in the same market (M2 and M12), it differed from its antimicrobial susceptibility patterns. Thus, no persistence was detected in the 36 markets evaluated during the study (Table 1).

STEC isolates harboring the* stx*1,* stx*1 plus* stx*2, and* stx*2 sequences, corresponded, respectively, to 5.3% (3/57), 8.8% (5/57), and 86.0% (49/57) (Table 2). Subtypes detected were* stx*2,* stx*2c-vha,* stx*2c-vhb, stx2/*stx*2c-vha, stx2/*stx*2c-vhb, and* stx*2c-vha/*stx*2c-vhb.

The* stx*2d variant was detected in 8 strains belonging to serotypes O2:H25, O82:H8, O130:H11, O174:H21, O178:H19 (2/13), ONT:H8, and ONT:H46 (Table 1). Their distribution was 7.7% (1/13) strain from HS, 16% (4/25) from MS, and 15.8% (3/19) from LS.

Among the O157 strains the prevalent genotype was* stx*2/*stx*2c-vha/*eae*/*exh*A detected in 54.5% (6/11) of strains, followed by the* stx*2/*eae*/*exh*A genotype in 27.3% (3/11) of strains. Among the STEC non-O157 strains, various genotypes were detected but the prevalent was* stx*2/*exh*A/*saa* in 30.4% (14/46).

Prevalence of adhesins was as follows:* eae* 26.3% (15/57) and* saa *24.6% (14/57). All the isolates belonging to O157, O26, ONT:H19, and OR:NM were attaching and effacing* E. coli *strains (*eae*+). Also,* ehx*A was detected in 61.4% (35/57) (Table 2) and expression of EHEC hemolysin calcium-dependent was observed among strains belonging to the following serotypes: O157:H7 (4/10), O157:NM, O8:H16, O8:H19, O15:H27 (1/2), O79:H19 (1/2), O82:H8, O113:H21 (1/2), O130:H11, O153:H21, O174:H28, O178:H19 (3/13), O179:H8, ONT:H7 (1/3), ONT:H8 (1/3), ONT:H19 (1/2), ONT:H21, ONT:H46, and OR:NT. As shown in Table 1, some strains belonging to the serotypes O26:H11, O157:H7 (6/10), ONT:H19 (1/2), and O178:H19 (1/10), although carrying the* ehx*A sequence did not exhibit enterohemolytic activity on blood agar plates supplemented with 10 mM CaCl_2_ (Table 1).

### 3.1. Antimicrobial Resistance

All the STEC isolates were susceptible to CIP, SXT, and C. Among the 57 STEC isolates, 16 (28.1%) were resistant to at least one of the antimicrobials examined in the first step (AMP, S, AK or TE). Eighteen (31.6%) strains showed reduced susceptibility. Three out eleven O157 strains were resistant to AMP (1), S (1), or TE and S (1). Table 1 shows reduced susceptibility to S, TE, or NA.

When the 11 AMP R/r STEC strains were phenotypically evaluated to detect *β*-lactamases, interactions between antimicrobials or zones compatible with ESBLs were not detected, but all the strains were R/r to KF. Carbapenemases were not detected.

Table 2 shows the distribution of virulence markers and antimicrobial resistance patterns of STEC strains by strata. Statistical analysis showed no significant differences in the frequencies among strata.

## 4. Discussion

The present study examined the (i) serotype (ii) virulence and (iii) antimicrobial profile of a geographical, socioeconomic, and temporal well-defined collection of STEC and revealed a high contamination of STEC O157 in low strata over time (71.4% of the sampling rounds) and the major antimicrobial resistance among strains at high strata. In our study,* stx *genes were detected in 91 out of 252 samples.

The present study was stratified by socioeconomic level due to the known association between the prevalence of childhood diarrhea and economic inequalities. Differences among socioeconomic groups are an important factor in the genesis of diseases related to the widespread microbiological contamination of the environment [32]. Several studies in Latin America have shown that adequate hygiene practices and good sanitary conditions decrease the levels of contamination significantly [14, 15, 33]. In our study, due to the sample size, no significant differences among strata were shown in the STEC contamination level of ground beef. Moreover, contamination persistence at the markets could not be detected. However, when the contamination of STEC O157 was considered independently, a seropathotype that has been associated to the 60% of HUS cases in Argentina [34] was prevalent in LS and MS, but only in LS over time (5 sampling rounds). This survey showed that, even though MS had the highest prevalence of STEC, the major risk of STEC O157 contamination was in LS.

The level of contamination by O157, confirmed by isolation, was 4.4% (11/252) slightly higher than former surveys (0.02–3.9%), carried out in ground beef, as well as in finished meat products, sausages, processed meat, and dairy products at different locations in Argentina [12, 13, 35–37].

Also, our results reached 36.1% of contamination of STEC, higher than those described by Gomez et al. in the city of Mar del Plata (8.4% of STEC) [38]. On the other hand, an increase in the level of contamination by STEC from 12.4% in carcasses to 25.0% at the markets reported by Etcheverría et al. could be due to cross contamination [14]. Likewise, Brusa et al. reported contamination of both meat and the environment (utensils and handler's hands) [15]. The results obtained in our study could not reveal that food contamination was the result of environmental contamination of the market, since the persistence was not demonstrated, but handlers as STEC carriers were not evaluated.

Our results showed that O178 and O157 are the prevalent STEC serogroups in ground beef. The serotype O178:H19 has been formerly isolated from other sources and clinical cases in Argentina [39–41] and Brazil [42].

The vast majority of outbreaks and sporadic cases in humans have been associated with serotype O157:H7; however, non-O157 serogroups are frequent over the world. In this study, serotypes O26:H11 and O113:H21 of international interest were isolated. It is important to highlight the detection of O26:H11 in food samples because it is an important pathogen in Argentina. Interestingly, the O145:NM serotype highly prevalent in Argentina was not isolated in the samples analyzed, probably due to the lack of enrichment, since IMS was only used for enrichment of O157, neither was the serotype O104:H4, which was responsible for the outbreak in Germany, isolated [43].

The four non-O157 serotypes prevalent in Argentina are O145:[H27,H-,NT], O121:[H19], O26:[H2,H11,NT], and O174:[H8,H21,H28,H-] [44]. Then, serogroup O174, which stands out as a local problem due to its clinical prevalence, is not considered in any European or American standard protocol. Currently, there is no specific diagnostic routine for O174. Despite this, 4 strains O174 were isolated in the present research and one of them was identified as* stx*2d.

According to virulence patterns of clinical strains from Argentina [45], STEC O157 strains isolated from ground beef showed the genetic profile* stx*2,* eae*,* ehx*A, but prevalent profiles of non-O157 varied. The phenotypic analysis showed that, similar to Feng et al., some strains that had* ehx*A did not exhibit enterohemolysis on blood agar plates supplemented with 10 mM CaCl_2_ [46].

Resistance (R) has been reported in* Enterobacteriaceae* and particularly in STEC [47–49]. Also, the multidrug resistance phenotype among diarrheagenic* E. coli* strains is emerging in different developing countries; for this reason the antibiotic resistance of the STEC isolates was investigated. Among the non-O157 strains we found only 1 isolate (332) R/r to 5 different antimicrobial families, but non-multidrug resistance (MR) based only on R results. In contrast, Mora et al. reported 92% of MR non-O157 from bovine and meat origin [50]. Also, Vidovic et al. detected MR phenotype in human and bovine isolates in Canada [51]. Published data for STEC isolates from foods, human, and veterinary sources indicated a tendency of increasing resistance rates to antibiotics [52]. Probably, the increase in resistance could be related to antimicrobial therapy or to the use of antimicrobials as growth promoters [51].

Most STEC isolates were resistant or showed reduced susceptibility to at least one of the common antimicrobial drugs AMP and/or S. The R to AMP could be inherent to the genus or the occurrence of extended-spectrum *β*-lactamase (ESBL) [30]. The high resistance R/r rate (59.6%) found in our study is similar to that reported in developing countries [53, 54] and constitutes a great concern in Argentina for public health. The resistance pattern most often observed was not the same as it was previously reported in Spain [50]. Unlike others, R to STX was not shown in our isolates [50, 51, 53, 54]. Opposite to Momtaz and Jamshidi, resistance of O26 serogroup was not detected [55]. Also, O113 serogroup showed only reduced susceptibility to S. The resistance was observed in all the socioeconomic strata. Therefore, STEC strains from ground beef, mainly from HS, may represent potential reservoirs of antimicrobial resistance genes as shown in Table 2.

In our study 8/15 attaching and effacing* E. coli* strains showed R/r, according to the reported association described by Buvens et al. [56]. According to Vidovic et al. the resistance to tetracycline detected in O157 strains was closer to bovine origin (36%) than to human one (4%) [51]. Considering that person-to-person transmission has been documented and the previous report of the same strain collected from handler's hands and meat samples in several markets, the animal or human origin from this tetracycline R strains must be considered [15, 57].

Indeed, 3/6 (50%) O157 R or r strains were obtained from LS, where poor hygienic measures were observed (data not shown). Momtaz and Jamshidi previously observed poor sanitation as primary factor responsible for the high antibiotic resistance in STEC from food [55].

STEC is a potential reservoir for antimicrobial resistance genes and plays an important role in the ecology of antimicrobial resistance of bacterial populations. Former reports demonstrated that the use of antimicrobial agents in farms is strongly associated with the prevalence of antimicrobial resistance in STEC strains isolated from food of animal origin. The genetic content, combination, and emergence frequency of this antibacterial susceptibility pattern may reflect the antibiotic selective pressure in this specific geographical region, providing useful antimicrobial surveillance information for the rational and effective use of antimicrobial agents [49].

The results of this study indicate that potentially pathogenic* E. coli *strains are widely distributed among ground beef of different socioeconomic levels. This is an interesting observation that should be taken into consideration for health intervention purposes.

The observed results imply the need to take good care in the slaughter of cattle, in the bromatological analysis of food at the markets, and in the techniques of disinfection and hygienic handling of foods, mainly ground beef.

Upcoming studies will allow (i) identifying the ability of isolates in producing biofilms, (ii) the presence of integrons that relate to the antibacterial susceptibility patterns described, and (iii) establishing the clonal relationship of the strains obtained from different sources.

## 5. Conclusion

In this study, by serial samples of ground beef sold at markets of different socioeconomic strata in a district of Argentina, a high level of STEC contamination was detected exceeding former reports. The virulence characteristics of the strains isolated in this study are those corresponding to strains with clinical impact in Argentina. Resistance patterns contribute to the strains characterization and to defining the possible epidemiological source.

STEC O157 detected in all the socioeconomic strata showed the spread of the pathogen and its risk to the population. According to the prevalence by strata, the risk was higher at low strata, where STEC O157 was recovered at several sampling rounds.

The immune capture of strains O26, O45, O103, O111, O121, and O145, with impact in USA and Europe, would not cover most of the common serogroups in several countries.

The STEC O178:H19 prevalent serotype, which was isolated without IMS in our study, suggests a higher contamination level than the recovered in this study. New diagnostic systems should be considered to identify these pathogens efficiently, according to the prevalence of the STEC seropathotypes in the country. Considering the high impact of HUS, it is necessary to establish more efficient microbiological analysis of food and provide the corresponding training in good hygienic practices to handlers to improve the quality of life of the population.

## Figures and Tables

**Table 1:** Characterization of Shiga toxin-producing *E.coli *according to their strata, market, and round of sampling.

Serotype	Virulence profile	*stx2* subtype	Strata market (sampling round)	R*	r**
O2:H25	*stx*2	*stx*2/*stx*2d***	M7 (6)	S	
O8:H16	Ehly, *ehx*A, *stx*1	—	M1 (5)		s
O8:H19	Ehly, *ehx*A, *stx*2, *stx*1, *saa *	*stx*2	M4 (2)		
O15:H27	Ehly, *ehx*A, *stx*2, *saa *	*stx*2	H8 (2)		
	*stx*2	*stx*2c-vhb	M1 (1)	AMP	s
O26:H11	*ehx*A, *eae*, *stx*1	—	M7 (6)		
O79:H19	Ehly, *ehx*A, *stx*2, *saa *	*stx*2	L6 (2)		
	Ehly, *ehx*A, *stx*2, *stx*1, *saa *	*stx*2	L11 (4)		
O82:H8	Ehly, *ehx*A, *stx*2, *saa *	*stx*2/*stx*2c-vhb/*stx*2d***	L8 (7)	S, AK	amp, cn, na, te, f
O113:H21	Ehly, *ehx*A, *stx*2, *saa *	*stx*2/*stx*2c-vhb	H11 (3)		s
	*stx*2	*stx*2c-vha	M7 (3)		s
O130:H11	Ehly, *ehx*A, *stx*2, *saa *	*stx*2c-vhb/*stx*2d***	L3 (4)		s
O153:H21	Ehly, *ehx*A, *stx*2	*stx*2	H8 (3)		s
O156:NM	*stx*2	*stx*2	H1 (2)	AMP	
O157:H7	Ehly, *ehx*A, *eae*, *stx*2, *stx*1	*stx*2c-vha	L9 (5)		na
	Ehly, *ehx*A, *eae*, *stx*2	*stx*2	H10 (2)	AMP	
		*stx*2/*stx*2c-vha	L1 (7); L7 (1)		
	*ehx*A, *eae*, *stx*2	*stx*2	L5 (7)		te
		*stx*2	M12 (2)****		
		*stx*2/*stx*2c-vha	M2 (7)****	S, TE	
			M12 (7)****	S	te
			L9 (6)		s
		*stx*2c-vha	M2 (5)****		
O157:NM	Ehly, *ehx*A, *eae*, *stx*2	*stx*2/*stx*2c-vha	M1 (2)		
O174:H21	*stx*2	*stx*2c-vhb/*stx*2d***	M11 (4)		s
		*stx*2c-vhb	L3 (3); L12 (5)		
O174:H28	Ehly, *ehx*A, *stx*2, *saa *	*stx*2	M11 (4)		s
O178:H19	Ehly, *ehx*A, *stx*2, *stx*1, *saa *	*stx*2c-vhb	H10 (1)		s
	Ehly, *ehx*A, *stx*2	*stx*2/*stx*2d***	M9 (1)		
		*stx*2c-vhb/*stx*2d***	H7 (3)		
	*ehx*A, *stx*2	*stx*2c-vha/vhb	M6 (1)		
	*stx*2	*stx*2c-vha/vhb	H1 (1)		
		*stx*2c-vha	H4 (2); M10 (5); L1 (2)	AMP	s
			M2 (4); M11 (4)		s
		*stx*2c-vhb	M7 (1)	AMP	s
			H5 (1); L7 (3)		s
O179:H8	Ehly, *ehx*A, *stx*2, *saa *	*stx*2	L9 (3)		s
ONT:H7	*α*-hemol, *ehx*A, *stx*2	*stx*2	M10 (1)	S	
	*stx*2	*stx*2/*stx*2c-vha	L10 (1)		
		*stx*2c-vha	H6 (6)	S	ak
ONT:H8	Ehly, *ehx*A, *stx*2, *saa *	*stx*2c-vhb/*stx*2d***	L3 (2)	AMP	s
	*stx*2	*stx*2	M7 (2)		
		*stx*2c-vha	M2 (4)		
ONT:H19	Ehly, *ehx*A, *eae*, *stx*2	*stx*2	L1 (7)	S	te
	*ehx*A, *eae*, *stx*2	*stx*2/*stx*2c-vha	M8 (6)		amp, s
ONT:H21	Ehly, *ehx*A, *stx*2, *saa *	*stx*2/*stx*2c-vhb	L3 (1)		
ONT:H46	Ehly, *ehx*A, *stx*2, *saa *	*stx*2c-vhb/*stx*2d***	M4 (5)		amp, s
ONT:NM	*stx*2	*stx*2c-vha	H11 (1)		
		*stx*2c-vhb	L1 (3)		
OR:NM	Ehly, *ehx*A, *eae*, *stx*1	—	M2 (2)		
ND	Ehly, *ehx*A, *stx*2, *stx*1, *saa *	*stx*2	H1 (7)	S	te

AMP, amp: ampicillin; AK, ak: amikacin; S, s: streptomycin; cn: gentamicin; Te, te: tetracycline; na: nalidixic acid; f: nitrofurantoin.

Socioeconomic strata: H: high, M: medium, L: low.

*R: antimicrobial resistance.

**r: reduced susceptibility.

***Mucus-activatable *stx*2d variant (Zheng et al. 2008) [10].

****Strains from different sampling rounds in the same markets (M2 (5, 7) and M12 (2, 7)) belonging to the same serotype showed differences by subtyping and antibiotyping.

**Table 2:** Distribution, according to the different socioeconomic strata, of virulence markers and antimicrobial susceptibility patterns of Shiga toxin-producing *Escherichia coli* isolated from ground beef in Buenos Aires, Argentina.

Strata	Proportion of STEC	Genotype						Phenotype						
		*stx*1	*stx*1 and *stx*2	*stx*2	*eae *	*saa *	*ehx*A	Serogroup		Antimicrobial susceptibility*				EHEC-Hly**
		*n* (%)	*n* (%)	*n* (%)	*n* (%)	*n* (%)	*n* (%)	O157	Non-O157***	S	R	R and r	r	*n* (%)
								*n* (%)	*n* (%)	*n* (%)	*n* (%)	*n* (%)	*n* (%)	
High	13/77	0 (0)	2 (13.3)	11 (84.6)	1 (7.7)	4 (30.8)	7 (53.8)	1 (7.7)	12 (92.3)	4 (30.8)	2 (13.3)	3 (23.1)	4 (30.8)	7 (53.8)
Media	25/91	3 (12)	1 (0.64)	21 (84.0)	8 (32.0)	3 (12)	15 (60.0)	5 (20.0)	20 (80.0)	10 (40.0)	3 (12)	4 (16.0)	8 (32.0)	7 (28.0)
Low	19/84	0 (0)	2 (10.5)	17 (89.5)	6 (31.6)	7 (36.8)	13 (68.4)	5 (26.3)	14 (73.7)	9 (47.4)	0 (0)	4 (21)	6 (31.6)	11 (57.9)

Total	57/252	3 (5.3)	5 (8.8)	49 (86.0)	15 (26.3)	14 (24.6)	35 (61.4)	11 (19.3)	46 (80.7)	23 (40.3)	5 (8.8)	11 (19.3)	18 (31.6)	25 (43.9)

*Susceptibility patterns: S: susceptible, R: resistant, r: intermediate or reduced susceptible strains.

**The alfa-hemolytic strain belonging to media strata is not included.

***Non-O157 include serogroups OR and ONT.
